# Harmaline to Human Mitochondrial Caseinolytic Serine Protease Activation for Pediatric Diffuse Intrinsic Pontine Glioma Treatment

**DOI:** 10.3390/ph17010135

**Published:** 2024-01-19

**Authors:** Morena Miciaccia, Francesca Rizzo, Antonella Centonze, Gianfranco Cavallaro, Marialessandra Contino, Domenico Armenise, Olga Maria Baldelli, Roberta Solidoro, Savina Ferorelli, Pasquale Scarcia, Gennaro Agrimi, Veronica Zingales, Elisa Cimetta, Simone Ronsisvalle, Federica Maria Sipala, Paola Loguercio Polosa, Cosimo Gianluca Fortuna, Maria Grazia Perrone, Antonio Scilimati

**Affiliations:** 1 Research Laboratory for Woman and Child Health, Department of Pharmacy-Pharmaceutical Sciences, University of Bari “Aldo Moro”, Via E. Orabona 4, 70125 Bari, Italy; morena.miciaccia@uniba.it (M.M.); antonella.centonze1@uniba.it (A.C.); domenico.armenise1@uniba.it (D.A.); olga.baldelli@uniba.it (O.M.B.); roberta.solidoro@uniba.it (R.S.); savina.ferorelli@uniba.it (S.F.); 2 Department of Biosciences, Biotechnologies, and Environment, University of Bari “Aldo Moro”, Via E. Orabona 4, 70125 Bari, Italy; francesca.rizzo1@uniba.it (F.R.); pasquale.scarcia@uniba.it (P.S.); gennaro.agrimi@uniba.it (G.A.); paolaannamaria.loguerciopolosa@uniba.it (P.L.P.); 3 Laboratory of Molecular Modelling and Heterocyclic Compounds ModHet, Department of Chemical Sciences, University of Catania, Viale Andrea Doria 6, 95125 Catania, Italy; gianfranco.cavallaro@phd.unict.it; 4 Department of Pharmacy-Pharmaceutical Sciences, University of Bari “Aldo Moro”, Via E. Orabona 4, 70125 Bari, Italy; marialessandra.contino@uniba.it; 5 Department of Industrial Engineering (DII), University of Padua, Via Marzolo 9, 35131 Padova, Italy; veronica.zingales@uv.es (V.Z.); elisa.cimetta@unipd.it (E.C.); 6 Department of Drug and Health Sciences, University of Catania, Viale Andrea Doria 6, 95125 Catania, Italy; s.ronsisvalle@unict.it (S.R.); federica.sipala@phd.unict.it (F.M.S.)

**Keywords:** harmaline, *h*ClpP activation, DIPG, molecular modeling

## Abstract

Diffuse intrinsic pontine glioma (DIPG), affecting children aged 4–7 years, is a rare, aggressive tumor that originates in the pons and then spreads to nearby tissue. DIPG is the leading cause of death for pediatric brain tumors due to its infiltrative nature and inoperability. Radiotherapy has only a palliative effect on stabilizing symptoms. *In silico* and preclinical studies identified ONC201 as a cytotoxic agent against some human cancer cell lines, including DIPG ones. A single-crystal X-ray analysis of the complex of the human mitochondrial caseinolytic serine protease type C (*h*ClpP) and ONC201 (PDB ID: 6DL7) allowed *h*ClpP to be identified as its main target. The hyperactivation of *h*ClpP causes damage to mitochondrial oxidative phosphorylation and cell death. In some DIPG patients receiving ONC201, an acquired resistance was observed. In this context, a wide program was initiated to discover original scaffolds for new *h*ClpP activators to treat ONC201-non-responding patients. Harmaline, a small molecule belonging to the chemical class of β-carboline, was identified through Fingerprints for Ligands and Proteins (FLAP), a structure-based virtual screening approach. Molecular dynamics simulations and a deep in vitro investigation showed interesting information on the interaction and activation of *h*ClpP by harmaline.

## 1. Introduction

Diffuse intrinsic pontine glioma (DIPG), primarily occurring in children [1], is a high-grade glioma (HGG) centered around the pons, belonging to the category of diffuse midline glioma (DMG), as recently classified by the WHO [2].

**Figure 1:**
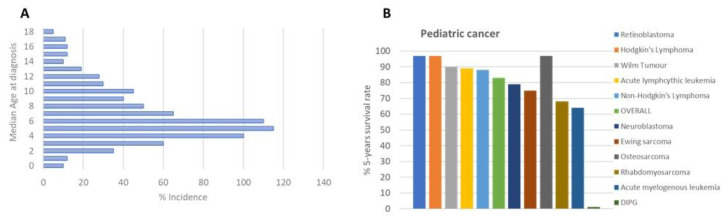
(**A**) Age of DIPG patients at diagnosis; (**B**) DIPG 5-year survival rate [https://www.dipg.org/dipg-stats accessed on 12 November 2023].

DIPG is the most common brainstem tumor in children, accounting for approximately 75–80% of all cases; 150–300 patients are diagnosed with DIPG in the USA per year, and a similar number occurs in Europe. The median age of patients with DIPG is approximately 4–7 years (Figure 1A). No gender difference has been observed among DIPG patients [2].

DIPG’s clinical treatment, like many other diseases, is borrowed from adults, with unsuccessful results despite extensive research efforts made in the field, as indicated by hundreds of publications, with more than 500 papers in the last six years. Radiation therapy is the first-line treatment for DIPG patients, with a survival benefit of only 3 months [3] (Figure 1B), transiently improving neurological deficits.

**Figure 2:**
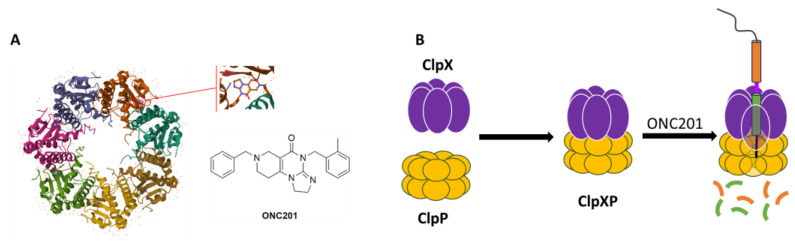
(**A**) Chemical structure of ONC201 and horizontal view of the complex between ONC201 and *h*ClpP (adapted from PDB ID: 6DL7); (**B**) structure of the ClpXP complex.

Most of the research on DIPG is performed with ONC201 (Figure 2A), an imipridone, which has raised hopes as its use in the treatment of glioblastomas in bevacizumab-resistant adults led to an increase in the overall survival (OS) and, in some cases, the regression of the primary thalamic lesion [4].

In May 2019, a diffractometric characterization (single-crystal X-ray) of the complex between the human mitochondrial caseinolytic serin-protease P (*h*ClpP) and ONC201 (PDB ID: 6DL7) (Figure 2A) led to the identification of this enzyme as its main ONC201 biological target [5].

*h*ClpP is part of a ClpXP mitochondrial matrix cylinder-shaped complex (*h*ClpP serine protease and the ATP-dependent proteases (AAA+ proteases) ClpX), which is an ATP-dependent protease that mediates the active remodeling, unfolding, and degradation of mitochondrial proteins using energy derived from ATP (Figure 2B). AAA+ proteases identify misfolded or damaged proteins in mitochondria [6]. Changes in cellular metabolism and bioenergetics, oxidative stress, and the intracellular level of ROS are hallmarks of cancer development, and these proteases are also important for the proliferation and metastasis of some types of cancers [7].

**Figure 3:**
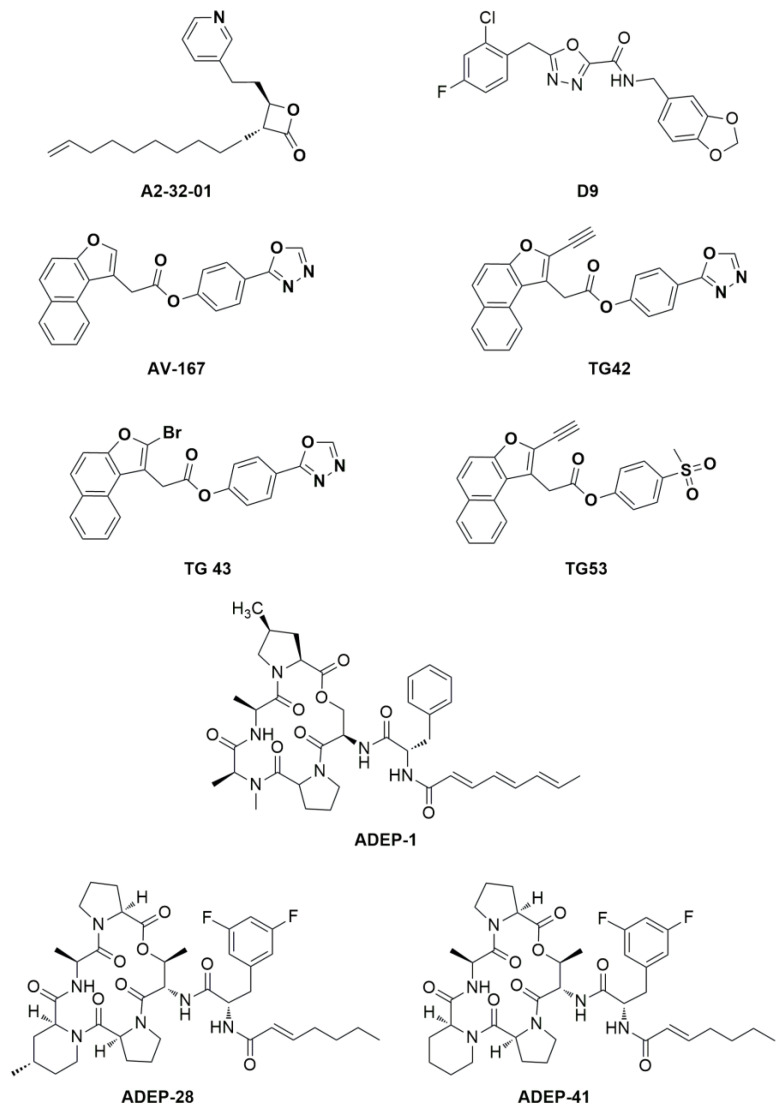
Chemical structure of *h*ClpP inhibitors and inducers.

Both the genetic and chemical inhibition of ClpXP and its overactivation by chemical and/or by mutation cause tumor cell death. In fact, on the one hand, its inhibition leads to the accumulation of misfolded and damaged respiratory chain proteins and impairs oxidative phosphorylation, resulting in the selective death of cancer cells [8]. Inhibitors of *h*ClpP covalently modify the fourteen catalytic residues of Ser153 located within the lumen of the *h*ClpP tetradecamer [9]. Several inhibitors of *h*ClpP have been developed, and their chemical structures have been optimized. Beta-lactones were initially developed as antibiotics to treat *Staphylococcus aureus* infections. A2-32-01 (Figure 3) also showed cytotoxicity in leukemic cell lines, osteosarcoma cell line 143B, and primary leukemic cells overexpressing *h*ClpP [7,10]. Despite the positive results in in vitro tests, the reduced plasma stability due to the easy lactone hydrolysis and their poor selectivity limited the corresponding clinical development [11].

The phenyl esters AV-167, TG42, TG43, and TG53 (Figure 3) were developed to improve the chemical stability and potency of β-lactones, but they exhibited off- and on-target effects that blocked any further study [9].

The overactivation of *h*ClpP by its activators results in mitochondrial morphological damage and a decrease in oxidative phosphorylation, inducing tumor cell death [8]. Therefore, targeting AAA+ proteases like ClpXP could be a strategy against malignant cells sparing normal tissues. Activators binding to the allosteric *h*ClpP site produce conformational changes in the enzyme complex, resulting in the enlargement of the axial proteolytic pore and the compaction of the protease. These changes lead to *h*ClpP hyperactivation and increase substrate degradation in a selective and uncontrolled manner [8]. To date, it is not known whether the inhibition or activation of *h*ClpP the preferred therapeutic strategy would be. 

Three classes of *h*ClpP activators are known: (1) acyldepsipeptides (ADEPs: the representative members of ADEPs are ADEP-1, ADEP-28, and ADEP-41 (Figure 3)) are antibiotics that bind to hydrophobic pockets present between neighboring *h*ClpP subunits, which usually bind the IGF loop of ClpX, inducing an enlargement of the axial pore and structuring of the axial *h*ClpP loop, resulting in unregulated entry and protein degradation [12,13]; (2) oxadiazocarboxyamides, like D9 (Figure 3), whose binding induces the dissociation of the ClpXP complex and the stabilization of the *h*ClpP activated state by widening the axial pore of the protease [14]; and (3) imipridones, like ONC201, activate *h*ClpP in a dose-dependent manner, as ADEP and D9 do [15]. ONC201 binds noncovalently to the hydrophobic allosteric pockets present between neighbouring subunits of *h*ClpP. Specifically, ONC201 binding induces *h*ClpP axial pore enlargement, while the complex adopts a more compact conformation like that induced by the binding of ADEP and D9 [16]. 

Despite the rapid development of ONC201, its pharmacokinetic and pharmacodynamic profiles need to be improved. In fact, the doses used in clinical trials are very high; not all patients respond to the treatment, and its antipsychotic effects should also be considered, as it acts as a dopamine D2 receptor (DRD2) antagonist [17]. 

Herein, a drug-repurposing study is described. It was accomplished starting with an *in silico* investigation using ~1500 natural products already on the market with other therapeutic indications, aimed at identifying a novel original scaffold to be used to build more performant *h*ClpP activators. Harmaline was identified by a structure-based virtual screening study using Fingerprints for Ligands and Proteins (FLAP). It was tested as a *h*ClpP activator, a cytotoxic compound on two representative DIPG cell lines (SU-DIPG-36 and SU-DIPG-50), spheroids of SH-SY-5Y and SK-N-AS, and an efflux pump substrate or modulator. Molecular dynamics experiments were used to explain the obtained results.

## 2. Materials and Methods

### 2.1. Computational Studies

A database of NPs was downloaded from INDOFINE Chemical Company (https://indofinechemical.com, accessed in 16 May 2022) and SPECS libraries (https://www.specs.net/index.php, accessed in 16 May 2022). This database consists of 1536 natural compounds, 1489 from the SPECS library and 47 from INDOFINE; these are all commercially easily available. They are NPs reflected in the pharmaceutical industry and have a specific activity. The *in silico* evaluation (PLS analysis) of the database was carried out using Volsurf+ (VS+) software (version 1.1.2, build date: 21 December 2016). Virtual screenings were performed through FLAP software (version 2.2.2, build date: 12 February 2020) in structure-based mode (SBVS) [18]. The software identifies the interaction fields (MIFs), calculated in GRID, which represent the interactions between the molecules under examination and the areas of interest (called pockets) identified within the crystalline structure [18,19]. GRID MIFs were generated using four molecular probes: H (shape, steric effects), DRY (hydrophobic interactions), N1 (H-bond donor), and O (H-bond acceptor) interactions. In addition, the SBVS mode returns three other important scores for evaluating interactions: GLOB-SUM, GLOB-PROD, and Distance. The first two values refer to the summation and production of the interactions, respectively. The Distance score represents the overall similarity derived from a combination of the degree of overlap between the individual probes (H, DRY, O, and N1) of the MIFs calculated for each candidate ligand and binding site. The crystallized structure used on FLAP is *h*ClpP in complex with ONC201 (PDB code: 6DL7; resolution, 2.00 Å). The GLOB-SUM score was used as a reference to evaluate the degree of interaction. The GLOB-PROD is not used because it is not very indicative; it could be influenced by interaction scores with zero values.

Molecular dynamics simulations were conducted using the optimal poses for harmaline and ONC201 within the pocket. Molecular dynamics studies were performed using Flare software version 6 (Cresset^®^, Litlington, Cambridgeshire, UK) [20,21].

The structures of the ligands were obtained from molecular docking studies and subsequently optimized using the software function “Flare preparation ligand” to minimize their energy. Protein preparation was performed using the “ProteinPrep” function of the software. This tool allows for the automated preparation of proteins for docking and molecular dynamics simulations. A TIP3P waterbox was produced, and its complexity was minimized. The force field used for the proteins was AMBER FF14SB, and AMBER GAFF2 was used for the ligands [22,23]. 

Three simulations were conducted over a period of 10 ns. The root-mean-square deviation (RMSD) evaluation at the 10 ns mark revealed no significant variations. The Flare 6.0 software was employed to visualize and examine the molecular dynamics simulations. Furthermore, the WaterSwap absolute binding free energy method was utilized to determine the binding free energies of the ligand–protein complexes investigated, employing a function available in the Flare software. The binding free energy was calculated using Bennett’s method, thermodynamic integration (TI), and free-energy perturbation (FEP). The binding free energy values were obtained by calculating the arithmetic mean of the energies determined using these methods.

### 2.2. Biochemical Studies. Plasmid Construction, hClpP Expression, and Affinity Purification 

To obtain the *h*ClpP construct suitable for protein expression, cDNA was engineered by PCR. The *h*ClpP cDNA (GenBank accession no. Z50853.1) coding sequence was deprived of the first 57 amino acids [24] corresponding to the mitochondrial targeting sequence MTS, and the ATG codon of the initiating methionine was added. At the 3′ end of the coding sequence, the TEV cleavage site, a V5 epitope, and a 6xHis tag were added [25,26], followed by a stop codon (Table 1). The construct was cloned into the isopropyl-1-thio-B-D-galactopyranoside (IPTG)-inducible expression vector pET-21b. The *h*ClpP/pET recombinant plasmid was transformed into *E. coli* BL21-CodonPlus (DE3)-RIL competent cells (Stratagene); plasmid DNA was isolated from positive clones, and the insert was verified by DNA sequencing. The amino acid sequence is shown in Table 1.

The recombinant protein was expressed in *E. coli* BL21-Codon Plus (DE3)-RIL cells. Briefly, a log-phase 250 mL culture (OD600~0.6), grown in Luria-Bertrani Broth (LB; 10 g/L of tryptone, 5 g/L of yeast extract, 10 g/L of NaCl) supplemented with 0.1 mg/mL of ampicillin, was induced with 0.5 mM isopropil-β-D-1-tiogalattopiranoside (IPTG, Sigma) for 22 h at 22 °C with shaking at 260 rpm.

**Figure 4:**
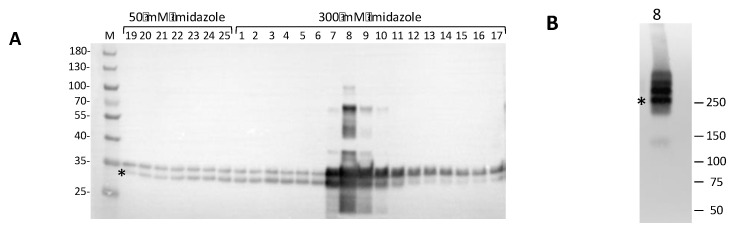
Western blot analysis of Ni-NTA eluted fractions. (**A**) Fractions eluted in 50 mM and 300 mM imidazole buffer were resolved on a 4–12% denaturing polyacrylamide gel (Bio-Rad Criterion XT Bis-Tris, Milan, Italy) in MOPS 1X buffer and blotted onto a PVDF membrane for Western blotting. A 5 µL sample of each fraction was loaded. Marker (M): PageRuler Prestained Protein Ladder (Thermo Fisher Scientific, Milan, Italy). *h*ClpP-specific bands were visualized by immunoreaction with polyclonal antiserum against *h*ClpP (1:20,000, ab124822, Abcam, Milan, Italy); (**B**) native PAGE/Western blotting of fraction n° 8 from the IMAC chromatography. The 5 µL samples were separated on a 4–15% protein gel (Bio-Rad Mini-PROTEAN TGX, Milan, Italy) in TGX 1X buffer. * has been positioned in correspondence to the *h*ClpP-specific bands. The positions and molecular masses of co-electrophoresed marker proteins are shown on the left (Precision Plus Dual Color Marker, Bio-Rad).

Bacteria were harvested by centrifugation at 4600× *g* for 15 min at +4 °C; pellets were washed with cold PBS and either immediately processed for protein extraction or fast frozen in liquid nitrogen and stored at −80 °C. To disrupt the bacteria, pellets were resuspended in 25 mL of lysis/binding buffer (25 mM of Tris/HCl pH 7,8, 150 mM of NaCl, 10% glycerol, 5 mM of imidazole); the mixture was added with 1 mg/mL of freshly prepared lysozyme (Sigma) in 10 mM of Tris/HCl with a pH of 8.0 and incubated on ice for 30 min with occasional swirling. The cells were sonicated on ice/water with Vibracell Sonics (large probe), a 30 s pulse followed by a 30 s pause at a 40% amplitude for ~60 min. Protein purification was achieved by immobilized metal ion affinity chromatography (IMAC) using a Ni-NTA prepacked column. After cell disruption, the cell lysate was cleared by ultracentrifugation at 100,000× *g* or, alternatively, by filtering through a 0.4 μm membrane. The NaCl concentration in all the buffers was low (150 mM) in order to preserve the double heptamer ring of the oligomeric *h*ClpP complex and, with it, the enzymatic activity. The S100 was passed through the IMAC column, and extensive washing was performed to remove most of the impurities, while the recombinant *h*ClpP protein remained bound due to the high affinity of the 6xHis tag. To improve *h*ClpP purification, two-step elutions were applied, one with a buffer containing 50 mM of imidazole, which removed most of the *E. coli* contaminating proteins, and the other at 300 mM of imidazole to elute the specifically bound protein. The identification of *h*ClpP in the eluted fractions was performed by an SDS-PAGE/Western blot analysis using a monoclonal antiserum against recombinant *h*ClpP. As shown in Figure 4A, all the eluted fractions revealed immunopositive bands. A typical profile of specific affinity elution was obtained in the fractions eluted with 300 mM of imidazole. In particular, fractions 7–10 showed several bands, two of which migrated with an apparent molecular mass between ~34 kDa and ~30 kDa. The calculated mass value for the recombinant *h*ClpP is 27.9 kDa, which is closer to the apparent size of the faster migrating band (asterisk). The slower migrating bands, which are more evident in fraction n° 8, could be likely caused by atypical forms of SDS–polypeptide complexes and/or partially denatured proteins, which often occur during gel separation [27,28]. Interestingly, a higher electrophoretic mobility for the human recombinant *h*ClpP separated by SDS-PAGE was also reported [29]. Fraction n° 8 from 300 mM imidazole elution, which was highly enriched in *h*ClpP, was also subjected to native PAGE. The *h*ClpP protein was revealed by Western blot analysis, showing three major bands (Figure 4B). Although it is difficult to predict the apparent molecular mass of the bands, one could speculate that the band migrating at ~250 kDa (the asterisk) might represent the heptamer ring. This would be in line with the finding that *h*ClpP expressed in *E. coli* forms a stable heptamer [30]. The bands with a higher apparent molecular mass could likely correspond to different oligomeric complexes, including the tetradecamer, which is in the enzymatic active form. 

For SDS-PAGE, column fractions (5 μL) were solubilized in 1x Laemmli buffer (Bio-Rad) and separated on a 4–12% denaturing polyacrylamide gel (Bio-Rad Criterion XT Bis-Tris) in MOPS 1X buffer. For native PAGE, a 5 μL volume of the indicated fraction was solubilized in 6X native protein loading buffer (600 mM of TrisHCl pH 6.8, 50% glycerol, 0.02% vromophenol blue without DTT) and separated on a 4–15% protein gel (Bio-Rad Mini-PROTEAN TGX) in TGX 1X buffer. For Western blotting, proteins in the gel were electrotransferred onto polyvinylidene difluoride (PVDF) membranes (immobilion-P PVDF membrane: 0.45 µm) at +4 °C for 3 h. Immunoblotting was performed according to standard techniques. The primary antibody was from Abcam; detection was performed with the HRP-conjugated secondary antibody (Bio-Rad). Chemiluminescent detection was achieved using Amersham ECL™ Prime Western blotting detection reagent (GE Healthcare Life Sciences, Marlborough, MA, USA) or Clarity Western ECL substrate (Bio-Rad, Hercules, CA, USA); signals were revealed by the ChemiDoc MP Imaging System (Bio-Rad, Hercules, CA, USA). Fractions containing *h*ClpP were stored on ice in a cold room for 24 h for the fluorogenic assay.

### 2.3. hClpP Activity Test

To assess the potency of the proteolytic activity of *h*ClpP, a FITC–casein assay was performed. In a black, flat-bottom, 96-well plate, a 3 μM solution of purified *h*ClpP in assay buffer (50 mM of HEPES, pH 7.5, 300 mM of KCl, 1 mM of DTT, 15% *v*/*v* glycerol; AB) preincubated at 37 °C for 15 min was added to 5 μL of the tested compounds in DMSO at different concentrations (ranging from 1 to 100 μM) and incubated for 15 min at 37 °C under shaking. As a control, three wells were filled with 5 µL of DMSO. The kinetic measurement was started after adding 2 µM of the fluorogenic peptides FITC and casein (Merck, C3777), used as hydrolytic substrates. The fluorescence of the cleaved FITC was recorded over 60 min at 37 °C on a Tecan Infinite M200 Pro (λ_ex_: 485 nm, emission λ_em_: 535 nm, gain: 60). The slope in the linear range between 600 and 1800 s was determined and plotted against time. The EC_50_ for each tested compound was calculated using GraphPad Prism 7.05. Results are reported as the mean of two independent experiments performed in triplicate.

### 2.4. Cell Cultures

Patient-derived diffuse intrinsic pontine glioma cell cultures (SU-DIPG-36, SU-DIPG-50) were provided by Dr Michelle Monje (Institutional Review Board (Stanford University) approval). The cells were cultured as a monolayer in media that was changed once a week at 37 °C in 5% CO_2_ by using Tumor Stem Media composed by: a 1:1 ratio of DMEM/F12 (Invitrogen)/Neurobasal (-A) (Invitrogen), B27 (-A) (Life Technologies, Milan, Italy), 20 ng/mL of human basic fibroblast growth factor (Life Technologies), 20 ng/mL of recombinant human epidermal growth factor (Life Technologies), 10 ng/mL of platelet-derived growth factor-AA, 10 ng/mL of platelet-derived growth factor-BB (Life Technologies), and 20 ng/mL of heparin (StemCell Technologies, Milan, Italy) [31,32,33].

Caco-2 and MDCK-BCRP cells were grown in Dulbecco’s high-glucose modified eagle medium, composed of 10% fetal bovine serum, 2 mM of glutamine, 100 U/mL of penicillin, and 0.1 mg/mL of streptomycin (all components purchased from Euroclone, Milan, Italy). Human neuroblastoma SH-SY5Y (ATCC-CRL-2266, MYCN not amplified) and SK-N-AS (ATCC CRL-2137, MYCN not amplified) cells (from Prof. E. Cimetta, Istituto di Ricerca Pediatrica Citta’ della Speranza, Padova, Italy) were cultured at 37 °C in 5% CO_2_ as a monolayer in high-glucose DMEM with an L-glutamine medium composed of 10% FBS, 1% MEM NEAA (100×), and 1% penicillin/streptomycin. The medium was replaced every 2–3 days. To obtain a single, centered, and highly reproducible spheroid per well, ultra-low attachment (ULA, Sarstedt^®^) 96-well round-bottom plates were used. The 3D spheroid cultures were generated from single-cell suspensions obtained from the trypsinized monolayers of both cell lines. Then, 200 μL of cell suspension (2 × 10^3^ cells) was seeded into each well of the plates. Each plate was centrifuged at 1200 rpm for 5 min to help the cells settle rapidly to the bottom of the wells. Each spheroid for both cell lines measured approximately 500 μm in diameter.

The 3D spheroids’ growth and morphology were monitored for seven days regarding changes in their volume and shape. Bright-field microscope images and morphological analyses of the spheroids were carried out with the inverted light microscope Zeiss Primo Vert equipped with a Zeiss camera (Axiocam 208 color, Zeiss Microscopy, Jena, Germany) at 10× magnification. The software Zen Lite version 2.6 (Zeiss Microscopy, Jena, Germany) was used to obtain their morphological parameters (diameter and area).

### 2.5. 2D Cell Viability Assay

Then, 1 × 10^4^ SU-DIPG-36 or SU-DIPG-50 cells/well in 100 µL of culture medium were seeded into 96-well plates and incubated O/N at 37 °C in CO_2_ (5%). Different concentrations of compounds (1–100 µM) were then added to the 100 µL of medium, and an incubation of 72 h followed. Control wells with DMSO were used to evaluate the possible cytotoxicity of the solvent. CCK8 (cat# 96992 Sigma Aldrich, Milan, Italy) (10 µL) was then added, and each plate was incubated for 3 h at 37 °C. Then, the absorbance was red (λ = 450 nm) with Tecan Infinite 200. GraphPad Prism allowed for the determination of IC_50_.

### 2.6. 3D Cell Viability Assay

Different concentrations of the tested compounds (12.5 to 100 μM) in 100 μL medium were added to the SH-SY5Y and SK-N-AS spheroids. The control wells contained solvent ≤1% *v*/*v*. The CellTiter-Glo^®^ 3D Luminescent Cell Viability Assay (Promega^®^, G968B, Milan, Italy) was used to measure intracellular ATP levels. After 72 h, the medium was removed, and CellTiter-Glo reagent (50 μL) was added. Cell lysis to extract ATP was performed by vigorous mixing (5 min). This was followed by 25 min of incubation at r.t. protected from light, transferring the supernatant to an opaque, white, flat-bottomed 96-well plate, and measuring luminescence using Tecan.

### 2.7. Calcein-AM Experiment 

Here, 3 × 10^4^ MDCK-MDR1 cells/well were seeded (100 µL) in a black 96-well culture plate and incubated in CO_2_ (5%) at 37 °C O/N. The compounds to be tested were added at different concentrations (0.1–100 µM) in 100 µL of culture medium and then incubated for 30 min. Then, 100 μL of calcein-AM (2.5 μM) in PBS was added to the plate, and another 30 min of incubation followed. Three washes with ice-cold PBS (100 µL) were performed, and the plate was read (λ_ex_ 485 nm and λ_em_ 535 nm) with Victor3 (PerkinElmer, Milan, Italy). The fluorescence of the wells containing the tested compounds was compared with that of those containing the untreated cells. The percentage of fluorescence as a function of log[dose] allowed the EC_50_ values to be determined [34].

### 2.8. Hoechst 33342 Experiment 

These experiments to measure the BCRP activity profile were conducted as described by Contino et al. [34], with minor modifications. Firstly, 3 × 10^4^ MDCK-BCRP cells/well were seeded (100 µL) in a 96-well black culture plate and brought to O/N confluence in a 5% CO_2_ incubator at 37 °C. Different concentrations (0.1–100 µM) of the compounds in the culture medium (100 µL) to be tested were added, and the plate was incubated for 30 min. Then, 100 µL of Hoechst 33342 in PBS was added to a final concentration of 8 µM and incubated for 30 min. The supernatant was aspirated, and the cells were fixed for 20 min with 4% PFA (100 µL), protecting them from light. Three washes with ice-cold PBS and three washes with saline buffer followed, and then the plate was analyzed (λ_ex_ 350 nm and λ_em_ 460 nm) by Victor3 (PerkinElmer). EC_50_ values were calculated from the percentage increase in fluorescence compared to log[dose].

### 2.9. ATPlite Assay 

Firstly, 2 × 10^4^ MDCK-MDR1 cells/well were seeded in 100 µL of complete medium in a 96-well plate [34]. The plate was kept O/N in a CO_2_ incubator at 37 °C, and after this time, the experiment started with the addition of the compounds to be tested at the different concentrations chosen in a volume of 100 µL of medium. This step was followed by 120 min of incubation in a humidified atmosphere with 5% CO_2_ at 37 °C. Then, 50 µL of mammalian cell lysis buffer was added, and the plate was shaken orbitally (5 min). Furthermore, 50 µL of substrate was added to all the wells, and the plate was stirred again (5 min) and then adapted to the dark (10 min). Then, the luminescence was read with the Victor3 (PerkinElmer).

### 2.10. Drug Transport Experiments

The experiment started with the preparation of the Caco-2 monolayer, which occurred by seeding the cells (20,000/well) in Millicell plates (Millipore, Milan, Italy). Its growth was followed for 21 days by changing the medium occasionally and measuring its transepithelial electrical resistance (TEER) daily using an epithelial voltohmmeter (Millicell-ERS) [35] until at least 1000 W was reached.

After 21 days, the plate was washed twice with Hank’s balanced salt solution (HBSS) (Invitrogen). After the second wash, the wells were filled with buffer, and the plate was kept at 37 °C for 30 min. After the incubation time, the HBSS buffer was replaced with the solutions of the compounds to be tested at a concentration of 1 × 10^−4^ M. The plates were placed in an incubator at 37 °C for 120 min. The apparent permeability (P_app_), in units of nm s^−1^, was calculated [34,35].

## 3. Results and Discussion

### 3.1. Computational Studies Based on the Fingerprints for Ligands and Proteins (FLAP) Algorithm

**Figure 5:**
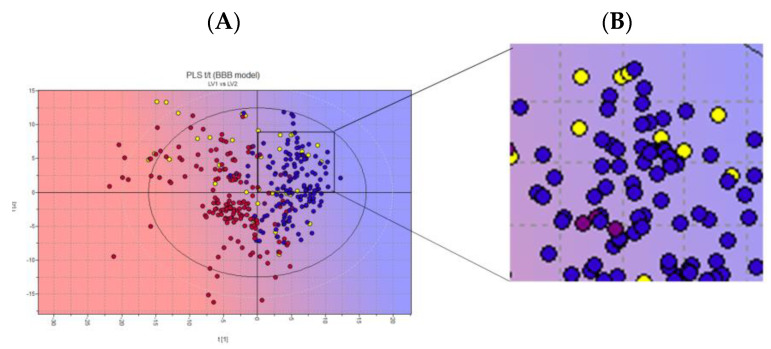
(**A**) Two-dimensional score plot of the PLS activity model for BBB crossing. (**B**) Zoomed-in image of chosen NPs. Projected compounds are colored according to their BBB permeability: red for low BBB permeability and blue for high; yellow dots: NPs database. Representative image of the INDOFINE Chemical Company library only.

In total, 1536 natural products (NPs) (INDOFINE Chemical Company and SPECS libraries) were subjected to an initial selection by a partial least-squares (PLS) analysis to predict their ability to cross the blood–brain barrier (BBB+). The entire database was projected onto a library model present in the software. According to the Volsurf analysis, the BBB+ compounds are in the blue area of the PLS region (Figure 5).

**Figure 6:**
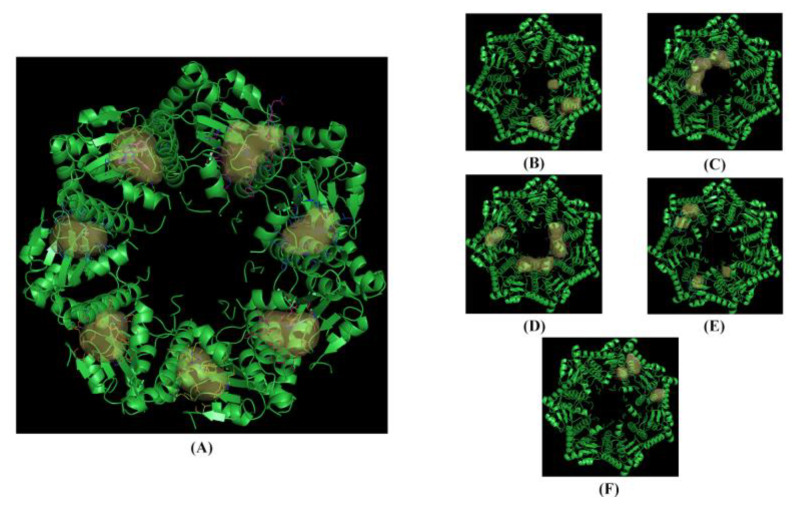
The crystallized structure and pockets of the *h*ClpP: ONC201 complex. (**A**) Front view, pockets 1 to 7; (**B**) back view, pockets 8 to 10; (**C**) back view, pocket 11; (**D**) back view, pockets 12 to 14; (**E**) back view, pockets 15 to 18; (**F**) back view, pockets 19 to 21.

The ten NPs selected (yellow dots in the box, Figure 4) were subjected to a structure-based virtual screening (SBVS) based on the Fingerprints for Ligands and Proteins (FLAP) algorithm. The SBVS was performed using the X-ray crystal structures of *h*ClpP in complex with ONC201 (PDB ID: 6DL7) [16]. The FLAP algorithm identified twenty-one pockets, seven of which were identical (pockets 1 to 7) (Figure 6). These seven pockets are equivalently repeated in the seven monomer units of the crystal structure, and within each of them, a molecule of ONC201 is present. Then, to study the interactions of *h*ClpP with the NPs under investigation, one of these seven pockets was randomly chosen.

**Figure 7:**
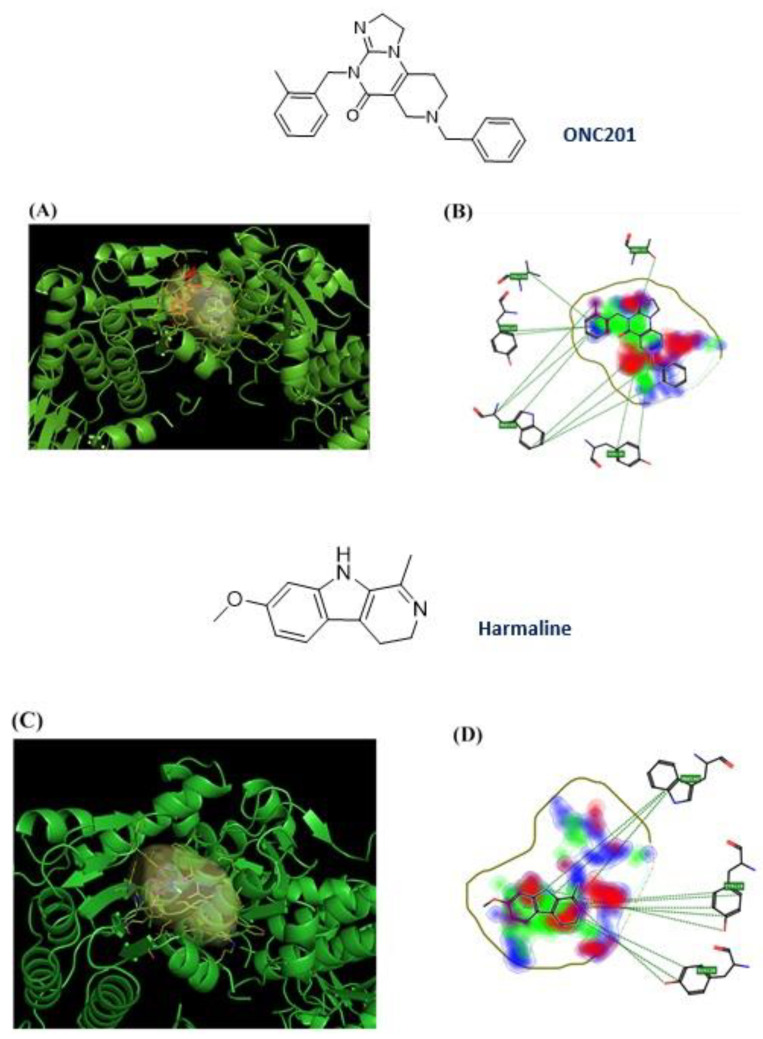
ONC201 and harmaline chemical structure and their 3D and 2D binding poses; (**A**) 3D binding poses for ONC201; (**B**) 2D binding pose for ONC201; (**C**) 3D binding poses for harmaline; (**D**) 2D binding poses for harmaline.

After the removal of the co-crystallized ONC201, the ten NPs were examined by the FLAP algorithm, and one of them, harmaline, showed an interaction score value similar to ONC201 (GLOB-SUM of 2.743 and 2.945, respectively). The respective 3D poses show similarities between the two compounds: the pocket area involved in the interaction is the upper one, and the two compounds are completely incorporated inside the pocket (Figure 6A–C). Similarities were also found in the 2D representation. The hydrophobic interactions (green areas) are the strongest in both cases, with small scattered areas of hydrogen bonding interactions (red and blue) (Figure 7B,D). The software highlights three redundant amino acid residues (Tyr118, Trp146, and Tyr138), which interact via hydrophobic interactions (π–π and CH–π) with the heterocycles. The compounds have similar structural features, such as a condensed heterocycle, which may have affected the virtual projections with FLAP (very similar scores and poses).

Harmaline (7-methoxy-1-methyl-4,9-dihydro-3*H*-pyrido[3,4-b]indole) is a naturally fluorescent (λ_ex_: 330 nm; λ_em_: 480 nm) [36] β-carboline alkaloid, isolated from *Peganum harmala* L., with a tricyclic pyrido[3,4-b]indole ring structure. It is the partially hydrogenated form of harmine [37,38].

**Figure 8:**
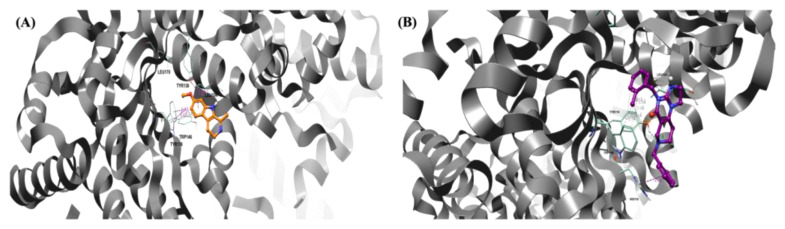
Pose of the two ligands in the pocket of *h*ClpP. Water and hydrogen were omitted for clarity. (**A**) Pose of harmaline (orange) in the pocket of *h*ClpP. The frame was captured at 8.2 ns. (**B**) Pose of ONC201 (magenta) in the pocket of *h*ClpP. The frame was captured at 6.5 ns.

Molecular dynamics studies have provided interesting results for harmaline. The molecule, characterized by its small structure and single rotatable bond, seeks stabilization during the initial nanoseconds of its dynamics, achieving a stabilized state during the final nanoseconds of the simulation, as illustrated in Figure 8A. In particular, the molecule endeavors to reach stability within the pocket for approximately 6 ns. Consequently, it forms hydrophobic (π–π) bonds with Tyr118, Tyr138, and Trp146, facilitated by the presence of three condensed rings. After approximately 8 ns, the molecule stabilizes in the selected pocket and moves slightly outward while simultaneously maintaining its π–π interaction with Trp146 and forming a hydrogen bond with Tyr138.

On the other hand, the dynamic simulations conducted on ONC201 (Figure 8B) revealed a good stability of the molecule within the pocket, with the formation of several hydrophobic interactions throughout the duration of the dynamics. It establishes π–π interactions with His116, Trp146, and Tyr138 throughout the duration of the simulation. Furthermore, the formation of a hydrogen bond with Tyr118 contributes to the stabilization of the molecule.

Overall, both molecules demonstrate stability within the pocket, which can be attributed to the diverse interactions they establish with the key amino acid residues proposed, Tyr118, Tyr138, and Trp146. Harmaline is characterized by three condensed rings and a reduced size compared with that of the reference molecule. This finding suggests that harmaline exhibits a reduced affinity for the examined pocket compared to ONC201 and displays notable but reduced stability.

As shown in Table 2, the binding free energies of ONC201 and harmaline obtained by the Waterswap method are −33.27 ± 1.64 and −27.91 ± 1.87, respectively. The observations from the binding free energy values are consistent with the visualization of the molecular dynamics simulations. Despite not demonstrating optimal free energy values, harmaline displays a sufficient binding affinity for *h*ClpP.

Harmaline has been extensively studied over the years for its broad pharmacological spectrum of activities. Indeed, besides its significant acetylcholinesterase (AChE) inhibitory activity with effects similar to the FDA-approved galantamine [39], it also inhibits monoamine oxidase [40] and myeloperoxidase activity [41].

**Figure 9:**
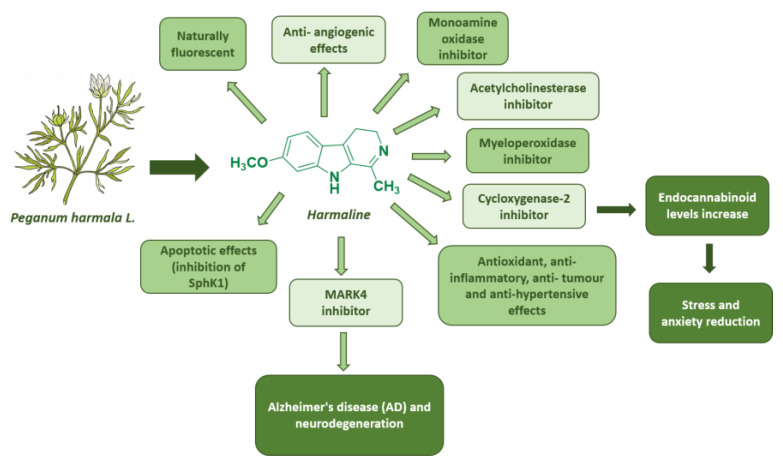
Summary of harmaline’s biological effects.

Harmaline also inhibits cycloxygenase-2 activity, resulting in increased endocannabinoid (EC) levels in the brain and a concomitant reduction in stress and anxiety [42]. Among harmaline’s numerous properties, its use results in an improvement in learning and memory disorders induced in mouse models by scopolamine and ethanol [43]. It also shows antioxidant, anti-inflammatory, anti-tumor, and antihypertensive effects [43]. Harmaline is cytotoxic in lung carcinoma cells by inhibiting sphingosine kinase-1/sphingosine-1-phosphate (SphK1), which is linked to cancer progression and the survival of cells affected by chemotherapy [44]. It also induces anti-angiogenic effects in mouse models of breast cancer [45]. Furthermore, a very recent study also suggested harmaline as a promising candidate to treat Alzheimer’s disease (AD) progression and neurodegeneration for its ability to inhibit microtubule affinity regulatory kinase 4 (MARK4), which is responsible for the development of cancer, diabetes, and neurodegenerative diseases [46] (Figure 9).

### 3.2. hClpP Activation by Harmaline and Anticancer Strategy

**Figure 10:**
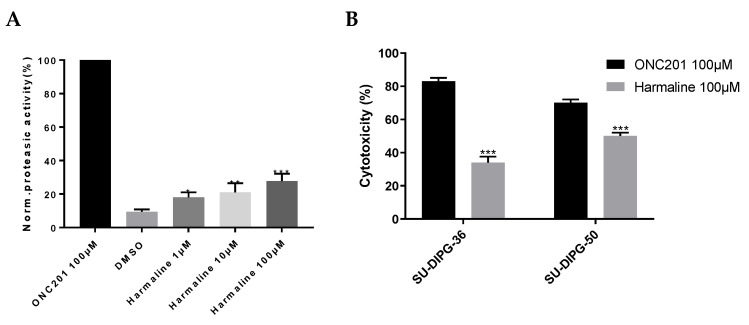
(**A**) *h*ClpP induction by harmaline at different concentrations normalized to 100 µM ONC201 activity. DMSO was used as a control. Each bar represents the mean ± SEM of three experiments performed in triplicate. A one-way ANOVA vs. DMSO was applied for * = *p* < 0.05; ** = *p* < 0.01; and *** = *p* < 0.0005; (**B**) cytotoxicity of 100 µM of ONC201 and 100 µM of harmaline on SU-DIPG-36 and SU-DIPG-50. Each bar represents the mean ± SEM of three experiments performed in triplicate. A two-way ANOVA was applied: SU-DIPG-36: ONC201 vs. harmaline at *p* = 0.001 and SU-DIPG-50: ONC201 vs. harmaline at *p* = 0.001.

To evaluate the *h*ClpP-activating potency of harmaline, an in vitro FITC–casein assay was performed. The mitochondrial *h*ClpP was bacterially expressed, purified, and used to test three scalar concentrations of harmaline (1, 10, 100 µM). A 28% activation of *h*ClpP was observed at the highest concentration, compared to the 100 µM of ONC201 used as a reference compound (Figure 10A).

Some malignancies have specific mitochondrial characteristics that make them dependent on oxidative phosphorylation (OXPHOS), the fundamental mitochondrial process linking the tricarboxylic acid (TCA) cycle for the synthesis of cellular building blocks such as aspartate and increasing ATP turnover. In these cases, OXPHOS is useful as a target to achieve a selective cytotoxic effect on malignant cells. Thus, targeting ClpP becomes an emerging antitumor strategy that takes advantage of the increased dependence of oxidative phosphorylation in these tumor types. Harmaline is known to exert its antitumor properties by modulating cellular and molecular mediators involved in cell cycle progression, cell proliferation, ROS balance, and apoptosis [47].

Therefore, the cytotoxic activity of harmaline was evaluated on two H3K27M-altered cell cultures derived from DIPG patients, SU-DIPG-36 and SU-DIPG-50 [48], and on neuroblastoma spheroids (SH-SY5Y and SK-N-AS).

Both DIPG cell lines have unamplified MYCN, a gene involved in tumor development and progression. Furthermore, SH-SY5Y is derived from a primary neuroblastoma, while SK-N-AS is from metastases [49,50,51].

After a 72 h exposure of SU-DIPG-36 and SU-DIPG-50 to 100 µM of harmaline, the measured cancer cell death was 34 and 50%, respectively. The high concentration of ONC201 shows a cytotoxic effect of 83 and 70% on the same cell lines, respectively (Figure 10B).

**Figure 11:**
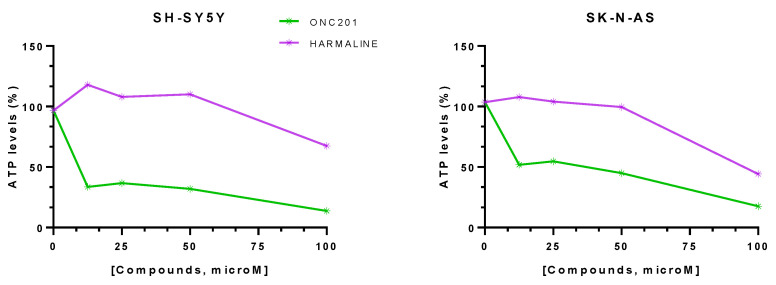
Percentage (%) ATP levels in SH-SY-5Y and SK-N-AS spheroids after 72 h of drug exposure. Data are expressed as mean ± SEM of two independent experiments (n = 2).

The mitochondrial toxicity on neuroblastoma cell lines was evaluated by an ATP assay after 72 h of exposure to different concentrations of compounds (12.5–100 μM) (Figure 11).

For both cell lines, the spheroids exposed to ONC201 revealed a decrease in cell viability of ~85% (Table 3). The highest harmaline concentration led to a decrease in cell viability for the SH-SY-5Y spheroids of ~32.5% and of ~56% for the SK-N-AS spheroids (Table 3).

### 3.3. Harmaline and Cell Efflux Pump Interactions

The effectiveness of the drug is also related to its intracellular turnover, which is mainly dependent on its activity towards the drug in the membrane efflux. It can be modified by the drug’s features with respect to a membrane efflux pump, such as P-glycoprotein (P-gp). P-gp is an efflux protein present at the apical level of the BBB, responsible for the efflux of endogenous and exogenous ligands out of the CNS [50], and is considered the BBB gatekeeper, our brain’s first line of defense. Thus, to predict the ability of a drug to overcome the BBB and hit central targets, measuring its interaction with P-gp is pivotal. A drug can be a substrate, modulator, or inhibitor of P-gp. Most known drugs are P-gp substrates. Three distinct biological assays are commonly used to evaluate the P-gp interacting mechanism: the calcein-AM assay, ATP consumption, and the apparent permeability (Papp) determination.

Since calcein-AM is a pro-fluorescent probe and a substrate of P-gp [51], it is effluxed from P-gp, remaining outside of cells that overexpress P-gp, such as BBB cells. When a compound interacts with P-gp, it can compete with the efflux of calcein-AM, allowing the probe to enter cells, where it is hydrolyzed by cytoplasmic esterases into the fluorescent calcein. Calcein, not being a substrate of P-gp and being hydrophilic, remains in the cell, giving a fluorescence signal. As evident from Table 4, no interaction was observed at the P-gp level for harmaline (EC_50_ > 100 μM), while a moderate interaction was observed for ONC201 (EC_50_ = 13.4 μM), potentially explaining the massive doses needed to produce effective action on patients. P-gp is ATP-dependent, so its substrates result in the cell depletion of ATP, while untransported compounds show an unchanged ATP cellular level. However, since we measured an interaction with P-gp but no associated consumption of ATP, we suggest that ONC201 could be included in the particular category of molecules interacting with P-gp, which are defined as non-transported [52].

### 3.4. Biological Membrane Permeability by Harmaline

The determination of P_app_ allows us to establish the permeability of harmaline and ONC201 both in the basolateral–apical direction (P_app_BA) and in the opposite direction (P_app_AB). P_app_ is measured using a monolayer of the human colon carcinoma cell line (Caco-2 cells). This cell line endogenously expresses a variety of transporter proteins present in various endothelial and epithelial barriers of the body; in particular, it expresses the P-gp and BCRP efflux pumps present at the BBB [53]. Since Caco-2 cells express P-gp only apically, fluxes across the monolayer in the basolateral–apical direction (P_app_BA) indicate passive transport. ONC201 and harmaline passively cross the membranes with a 2157 and 2539 nm/s flux, respectively. Active, mediated efflux is represented by the P_app_AB value. The residual flow of ONC201 of 356 nm/s is due to the fact that the drug is retained by P-gp (EC_50_ = 13.4 µM). Harmaline’s P_app_AB is 496 nm/s, but given its P-gp EC_50_ > 100 µM, its possible interaction with another efflux pump normally expressed at the apical level (BCRP) was studied. Thus, the BCRP activity for harmaline was measured, and its EC_50_ was found to be 14.9 μM.

## 4. Conclusions

This work arises from the need to identify new enhancers of *h*ClpP activity. Then, using the FLAP algorithm, harmaline was identified as a compound with similar features to ONC201. The outcomes of the molecular dynamics simulations demonstrated reduced stabilization in the harmaline binding site in contrast to ONC201; however, encouraging interactions between harmaline and *h*ClpP were still observed. Harmaline has been shown to be a moderate enhancer of *h*ClpP activity with moderate cytotoxic activity against both DIPG and neuroblastoma cells, which share the non-amplified MYCN gene involved in tumor onset and progression. It was interesting to verify how harmaline crosses the blood–brain barrier through a combination of three in vitro tests, and this led to the hypothesis that the efflux pump involved in its transport is BCRP. The results will allow us to start research based on specific modifications of the harmaline chemical structure, which will lead to structure–activity relationship investigations aiming to identify compounds more potent than ONC201 and, therefore, capable of really contributing to fighting DIPG.

## Figures and Tables

**Table 1:** *h*ClpP amino acid sequence.

MPLIPIVVEQTGRGERAYDIYSRLLRERIVCVMGPIDDSVASLVIAQLLFLQSESNKKPIHMYINSPGGVVTAGLAIYDTMQYILNPICTWCVGQAASMGSLLLAAGTPGMRHSLPNSRIMIHQPSGGARGQATDIAIQAEEIMKLKKQLYNIYAKHTKQSLQVIESAMERDRYMSPMEAQEFGILDKVLVHPPQDGEDEPTLVQKEPVEAAPAAEPVPASTENLYFQGKLGKPIPNPLLGLDSTRTGHHHHHH	
TEV sequence	ENLYFQG
V5 epitope	GKPIPNPLLGLDST
His Tag	HHHHHH

**Table 2:** Binding free energies of ONC201 and harmaline with *h*ClpP calculated by Bennet, TI, and FEP methods and relative consensus average.

Ligand	Bennet (kcal/mol)	FEP (kcal/mol)	TI (kcal/mol)	Consensus (kcal/mol)
Harmaline	−28.13	−28.08	−27.49	−27.91 ± 1.87
ONC201	−34.08	−32.51	−32.44	−33.27 ± 1.64

**Table 3:** ONC201’s and harmaline’s (100 µM) mitochondrial toxicity on SH-SY-5Y and SK-N-AS spheroids measured by ATP assay after 72 h of exposure to drugs. Data are expressed as mean ± SEM of three independent experiments (n = 3).

Drugs	SH-SY-5Y Cell Viability (%)	SK-N-AS Cell Viability (%)
ONC201	13.67 ± 6.23	17.5 ± 6.80
Harmaline	67.48 ± 13.91	44.24 ± 24.25

**Table 4:** Tested compounds’ efflux pump activity and apparent permeability (P_app_) values. Data are expressed as the mean of three independent experiments; NT = not tested.

Drugs	P-gp EC_50_, µM	ATP	P*_app_*BA (nm/s)	P*_app_*AB (nm/s)	BCRP EC_50_, µM
ONC201	13.4	NO	2157	356	>100
Harmaline	>100	NT	2539	496	14.9

## Data Availability

Data are contained within the article.
